# Movement priming of EEG/MEG brain responses for action-words characterizes the link between language and action

**DOI:** 10.1016/j.cortex.2015.10.021

**Published:** 2016-01

**Authors:** Giovanna Mollo, Friedemann Pulvermüller, Olaf Hauk

**Affiliations:** a Medical Research Council Cognition and Brain Sciences Unit, Cambridge, UK; b University of York, Department of Psychology, York, UK; c Institute for Advanced Biomedical Technologies, G D'Annunzio University, Chieti, Italy; d Brain Language Laboratory, Department of Philosophy, Freie Universität Berlin, Germany

**Keywords:** Embodiment, Semantics, Semantic somatotopy, Word recognition, Motor cortex

## Abstract

Activation in sensorimotor areas of the brain following perception of linguistic stimuli referring to objects and actions has been interpreted as evidence for strong theories of embodied semantics. Although a large number of studies have demonstrated this “language-to-action” link, important questions about how activation in the sensorimotor system affects language performance (“action-to-language” link) are yet unanswered. As several authors have recently pointed out, the debate should move away from an “embodied or not” focus, and rather aim to characterize the functional contributions of sensorimotor systems to language processing in more detail. For this purpose, we here introduce a novel movement priming paradigm in combination with electro- and magnetoencephalography (EEG/MEG), which allows investigating effects of motor cortex pre-activation on the spatio-temporal dynamics of action-word evoked brain activation. Participants initiated experimental trials by either finger- or foot-movements before executing a two alternative forced choice task employing action-words. We found differential brain activation during the early stages of subsequent hand- and leg-related word processing, respectively, albeit in the absence of behavioral effects. Distributed source estimation based on combined EEG/MEG measurements revealed that congruency effects between effector type used for response initiation (hand or foot) and action-word category (hand- or foot-related) occurred not only in motor cortex, but also in a classical language comprehension area, posterior superior temporal cortex, already 150 msec after the visual presentation of the word stimulus. This suggests that pre-activation of hand- and leg-motor networks may differentially facilitate the ignition of semantic cell assemblies for hand- and leg-related words, respectively. Our results demonstrate the usefulness of movement priming in combination with neuroimaging to functionally characterize the link between language and sensorimotor systems.

## 1 Introduction

Embodied theories of semantics, proposing that semantic information is at least partly stored in distributed neuronal networks including sensory and motor systems of the brain, have recently gained support from cognitive neuroscience (Barsalou et al., 2003, Kiefer et al., 2008, Martin, 2007, Pulvermüller, 2001) as well as behavioral research (Boulenger et al., 2006, Glenberg and Kaschak, 2002, Meteyard et al., 2008). However, there is still controversy as to how exactly sensory-motor systems contribute to semantic processing, and whether the existing empirical evidence really is support for an “embodied” view on language (Chatterjee, 2010, Mahon and Caramazza, 2008). The empirical evidence is still ambiguous and inconsistent (see e.g., Hauk & Tschentscher, 2013). It is ambiguous in so far as behavioral interactions and brain activation usually only provide correlational rather than causal evidence, i.e., they demonstrate that processing action- or object-related stimuli affects sensorimotor systems, but not that activations in these areas affect language processing. It is inconsistent in so far as the pattern of activation in functional magnetic resonance imaging (fMRI) studies varies across studies (Postle, McMahon, Ashton, Meredith, & de Zubicaray, 2008), some studies have found effects of transcranial magnetic stimulation (TMS) stimulation on action-word processing (Kuipers et al., 2013, Pulvermüller et al., 2005a) while others have not (Tomasino, Fink, Sparing, Dafotakis, & Weiss, 2008), and some studies have found effects of lesions in motor areas on action concept processing (Kemmerer, Rudrauf, Manzel, & Tranel, 2012) while others have not (Arevalo, Baldo & Dronkers, 2012). On this background, some authors have suggested that a debate “Embodied – yes or no?” is not meaningful, and should rather be framed as “Embodied – to what degree?” (Chatterjee, 2010, Hauk and Tschentscher, 2013).

While studies that focus on embodied semantics usually highlight the role of sensorimotor brain areas, it is usually assumed that these areas function in connection with other “core language areas” or “hubs” (Patterson et al., 2007, Pulvermüller et al., 2010, Rogers et al., 2004). The relative contribution of these poly- or a-modal regions on the one hand and the distributed modal regions on the other determine to which degree language processing can be considered as embodied. It is therefore important to characterize the link between sensorimotor and core language areas in more detail, and in particular its relationship to language performance. In order to specify the functional contributions of sensorimotor systems to semantic processing, we need experimental paradigms that allow us to show that

1) effects occur at a semantic level of processing [and not for example at a “post-translational stage” (Glenberg & Kaschak, 2002), as may be the case for metabolic imaging methods with low temporal resolution such as fMRI]
2) activation in sensorimotor systems affects language processing (rather than vice versa, as in neuroimaging studies that show sensorimotor activation during word processing)

We here propose to combine motor priming with EEG/MEG methodology to address these issues. In our experiment, experimental trials were initiated by finger or foot button presses, respectively. Participants kept a button pressed, and therefore the corresponding muscles flexed and motor cortex activated, while they were presented with an arm- or leg-related word. This allowed us to study the spatio-temporal brain dynamics during processing of different types of action-words while different parts of motor cortex were pre-activated. Furthermore, participants had to perform lexical and semantic Go/NoGo decisions at the end of each trial by releasing the button, thus offering the possibility to detect behavioral movement priming effects.

With respect to point 1) above, the high temporal resolution of EEG/MEG allows monitoring brain activity at latencies that have been associated with earliest stages of semantic information retrieval, e.g., around 150 msec after word presentation (Amsel et al., 2013, Hauk et al., 2012, Moseley et al., 2013, Pulvermüller et al., 2009). The exact time course of semantic processing is still debated, and the boundary between early lexico-semantic processing and later imagery or mental simulation processes may be fuzzy (Barsalou, 2009), but effects at such early latencies would rule out a “post-translational” explanation. Furthermore, state-of-the-art source estimation of combined EEG/MEG data – although not as precise as fMRI localization – allows the dissociation of effects in different language-related brain areas, such as inferior frontal lobe (“Broca's area”), anterior temporal lobe (ATL), and posterior superior temporal lobe (pSTL, “Wernicke's area”).

With respect to point 2), we propose to study effects of pre-activation of a specific effector or movement type (e.g., left/right finger/foot) on word processing. Thus, rather than showing that word processing activates motor areas, we can potentially reveal whether the activation state of the motor system affects language processing. Similar paradigms have already been used in behavioral and event-related potential (ERP) research (Boulenger et al., 2006, Dalla Volta et al., 2009, Glenberg et al., 2010, Helbig et al., 2010, Liepelt et al., 2012, Sato et al., 2008, Shebani and Pulvermüller, 2013, Van Elk et al., 2008, Van Elk et al., 2010). In our approach, we can study the effect of motor priming on both behavior and spatio-temporal brain dynamics.

In principle, this paradigm could also be applied to other stimulus modalities, e.g., using color priming of color words. The motor system is of particular interest, since it allows precise hypotheses about somatotopic activation patterns, and because motor cortex is relatively easy to study using different types of neuroimaging methods such as fMRI, electro- and magnetoencephalography (EEG/MEG), and TMS. fMRI studies have reported that stimuli referring to actions performed with different body parts elicit a somatotopic activation pattern within motor and premotor areas (e.g., “kick” or “pick” in leg- and hand-motor cortex, respectively) (Aziz-Zadeh et al., 2006, Boulenger et al., 2009, Buccino et al., 2001, Hauk et al., 2004, Kemmerer et al., 2008, Raposo et al., 2009, Tettamanti et al., 2005, Willems et al., 2010). EEG/MEG studies have provided evidence that differential action-word activation occurs early on in processing, around 150–200 msec after stimulus onset (Boulenger, Shtyrov & Pulvermuller, 2012, Hauk and Pulvermüller, 2004, Hauk et al., 2008, Kiefer et al., 2008, Klepp et al., 2014, Moseley et al., 2013, Pulvermüller, 2001).

TMS also enables the investigation of motor cortex activation on behavioral and EEG responses, with reasonable spatial and temporal resolution (Devlin & Watkins, 2007). However, TMS is limited to cortical sites close to the skull, and sampling of multiple stimulation latencies is cumbersome and may require between-subject designs. Importantly, one cannot generally assume a one-to-one relationship between brain regions and processes. Even if TMS stimulation of motor areas interferes with movement execution, it is still possible that sub-populations of neurons in the same area are involved in other cognitive functions than movement execution (e.g., sequence monitoring, attention to action etc.). An effect of TMS stimulation in such an area can therefore not uniquely be interpreted as a result of the disruption of specific movement execution processes.

A previous single-pulse TMS study found facilitation of arm- and leg-word processing after hand- and leg-motor cortex stimulation, respectively (Pulvermüller, Hauk, Nikulin, & Ilmoniemi, 2005). Another study only found TMS effects on action-word processing for pulses delivered at late latencies, and only in a task that encouraged motor imagery (Tomasino et al., 2008). A recent study reported disruption of semantic priming on the late N400 ERP component following rTMS stimulation of hand-motor cortex for hand-related but not mouth-related words (Kuipers et al., 2013). Thus, evidence for effects of motor cortex pre-activation on word processing is still scarce and inconsistent. Here, we propose a novel experimental paradigm that allows the assessment of effects of motor cortex pre-activation on brain dynamics and behavior.

Semantic priming effects, i.e., facilitated behavioral responses and reduced brain responses to stimuli following semantically related stimuli, are well-established in the literature (McNamara, 2005). A number of neural and cognitive mechanisms have been suggested to account for these and related findings (Henson & Rugg, 2003), including recent theories of predictive coding (Bastos et al., 2012, Summerfield and de Lange, 2014). Adapted to our paradigm, the trial-initiating movement takes the role of the semantic prime. Thus, we hold the view that in all these frameworks one would predict faster responses and lower brain activation for congruent compared to incongruent movement primes and word targets, e.g., for arm-words compared to leg-words following a finger button press. The somatotopy of action-words (SAW) model (Pulvermüller, 2005) suggests a mechanistic explanation in terms of cell assemblies. According to the SAW model, action-word semantics is stored in distributed cell assemblies comprising core language areas in left perisylvian cortex, as well as effector-specific parts of the sensorimotor cortex. These cell assemblies are supposed to have formed based on Hebbian principles (Hebb, Lambert, & Tucker, 1971), and ignition of one of its parts may trigger – or at least facilitate – the activation of the whole assembly.

Therefore, we here expect prime movements to facilitate responses to congruent compared to incongruent action-words. Importantly, we are also able to test for congruency effects in motor and non-motor brain regions at the earliest stages of semantic information retrieval. If action-word semantics is stored in cell assemblies including specific parts of motor cortex together with perisylvian language areas, then pre-activating the corresponding specific part of motor cortex should make it easier to ignite the whole assembly when the action-word is presented. Thus, we expect lower activation for congruent compared to incongruent stimulus pairs in perisylvian brain areas involved in early semantic information retrieval. A recent MEG study has reported differential activation for action-word types in motor areas around 150 msec after word onset (Moseley et al., 2013), and recent behavioral as well as ERP data have indicated that semantic information retrieval can begin already around 160 msec (Amsel et al., 2013, Hauk et al., 2012). Thus, congruency effects in perisylvian language areas in this latency range would be the strongest evidence for an effect of activity in motor cortex on language areas during semantic information retrieval.

EEG and MEG brain responses were measured simultaneously and combined for distributed source estimation. In a region-of-interest (ROI) analysis, we tested whether the congruency between effector type (i.e., hand or foot used for button presses) and word type (i.e., arm- or leg-related words) would modulate activity in left-hemispheric motor and language brain areas at early stages of processing. We selected language areas that have previously been implicated in semantic processing (Binder et al., 2009, Bookheimer, 2002, Geschwind, 1970, Patterson et al., 2007, Vigneau et al., 2006), and that we consider to be separable based on EEG/MEG source estimation, namely the ATL, the left pSTL, as well as inferior frontal gyrus (IFG).

## 2 Materials and methods

### 2.1 Participants

17 healthy native speakers of English entered the final analysis (10 females). Their mean age was 24 years (SD 5 years). All of them were right-handed according to a simplified version of Oldfield's handedness inventory (Oldfield, 1971), they had normal or corrected-to-normal vision and reported no history of psychiatric or neurological disease, head trauma, substance abuse or other serious medical conditions. Informed consent was obtained from all subjects and they were paid for their participation. This study was approved by the Cambridge Psychology Research Ethics Committee. None of the participants was aware of the purpose of the study at the beginning of the experiment.

### 2.2 Stimulus materials

The stimulus set included a) 40 arm-related words (e.g., “write”), b) 40 leg-related words (e.g., “kick”), as well as c) 80 abstract words (e.g., “hope”, for the semantic task) and d) 80 orthographically legal pseudowords (for the lexical decision task) (see Appendix for list of stimuli). Stimulus sets were matched for word length (a,b,c,d – referring to stimulus categories in previous sentence), word frequency (a,b,c), orthographic neighborhood size (Coltheart's N) (Coltheart, Jonasson, Davelaar, & Besner, 1977) (a,b,c,d), bi-gram and tri-gram frequency (a,b,c,d). Psycholinguistics parameters were obtained from CELEX lexical database (Baayen, Davidson, & Bates, 1993). The matching procedure was performed using the software match (Van Casteren & Davis, 2007) and the matching results are shown in Table 1. Note that only arm- and leg-related words entered the relevant analyses. All pseudowords were in accord with the phonological and orthographic rules of British English. The same action-related words were used for the four sessions of the experiment.

### 2.3 Procedure and experimental design

**Fig. 1:**
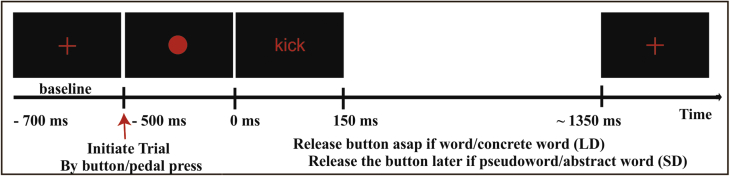
Trial structure for lexical decision (LD) and semantic decision (SD) tasks. Trials could be initiated by pressing a button either by finger or by foot. The “release” instructions refer to the lexical decision (LD, word/pseudoword) or semantic decision (SD, concrete/abstract) tasks, respectively. Target word categories in both tasks were arm-related (e.g., “stir”) and leg-related (“kick”) words.

The stimuli detailed above were used in a lexical decision task (LD) and a semantic decision (SD) task. Participants sat in a comfortable chair located in a dimly lit and sound-attenuated magnetically shielded room. The experiment was implemented with e-Prime 2.0 (Psychology Software Tools, Inc). Stimuli were written in white lowercase Arial font with size 24 on a black background and presented on a screen placed at 1.5 m in front of the participant. The size of each stimulus did not exceed a visual angle of 4°. Each trial began whit a white fixation cross (‘+’) presented for a random duration between 900 and 1500 msec, followed by a red dot which remained on the screen until the subject pressed the button. After a fixed period of 500 msec after the button press, a letter string appeared for 150 msec. Each trial ended with the presentation of a red cross after 1 sec (Fig. 1).

In LD the participants were instructed to press the button after the red dot presentation, keep it pressed, and wait for the presentation of the letter string. They had to release the button as quickly as possible in response to a word, but wait for the presentation of the red cross to release the button after a pseudoword presentation. In SD they followed a similar procedure, but had to release the button as quickly as possible after a concrete word (regardless of word type) and to wait for the presentation of the red cross to release the button after an abstract word.

In separate sessions, responses were given using the right index finger or the right foot. The sequence of task and response effector blocks was counterbalanced across subjects. Initially, each participant practiced each task until they felt comfortable with it.

Finger button presses were recorded using a conventional MEG-compatible button box. The participants rested their arms comfortably on a cushion, and pressed the corresponding buttons with little effort. Keeping a button pressed required sustained tension in the corresponding finger. Foot button presses were recorded using a purpose-made foot pedal, consisting of a button box inserted into the sole of a sandal. Buttons could be pressed by a small comfortable movement of the toes (the big toe executing the button press). As with the finger button press, keeping the button pressed required sustained tension in the toes.

### 2.4 Data acquisition

Electroencephalographic (EEG) and magnetoencephalographic (MEG) data were simultaneously collected in the magnetically and acoustically shielded room at the MRC Cognition and Brain Sciences Unit in Cambridge, UK. The EEG was recorded using 70 Ag/AgCl electrodes mounted on an electrode cap (Easycap, Falk Minow Services, Herrsching-Breitbrunn, Germany) placed according to the extended 10/20 system. The nose electrode was used as recording reference, while the ground electrode was placed on the left cheek. MEG signals were collected with a whole-head 306 channel Neuromag Vectorview system (Elekta AB, Stockholm) combining 204 planar gradiometers and 102 magnetometers. Data were sampled at 1000 Hz with a band-pass filter .03–333 Hz (MEG) and .1–333 Hz (EEG). The EEG had a gain of 5000 (1 mV maximum peak-to-peak signal to electrode input). Eye-movements and blinks were monitored using the electrooculogram (EOG) recorded bipolarly through electrodes placed above and below the left eye (vertical) and at the outer canthi (horizontal). The participants' head shapes were digitally collected with a 3Space Isotrak II System (Fastrak Polhemus, Colchester, VA), along with the position of five Head Position Indicator (HPI) coils, three anatomical landmark points (nasion, left and right preauricular points) and additional randomly distributed points on the head.

High-resolution structural T1-weighted magnetic resonance imaging (MRI) images were acquired in a 3T Siemens Tim Trio scanner at the MRC CBU, using a 3D MPRAGE sequence (field-of-view 256 mm × 240 mm × 160 mm, matrix dimensions 256 × 240 × 160, 1 mm isotropic resolution, TR = 2250 msec, TI = 900 msec, TE = 2.99 msec, flip angle 9°).

### 2.5 Data processing

We tested 24 native English speakers. One participant's data were discarded because of an exceptionally high error rate; six further participants' data were removed because of the high frequency of ocular artifacts (mostly eye blinks following trial initiation, and due to technical problems. Thus, data from 17 participants entered the final analysis.

### 2.6 Behavioral data processing

Behavioral data were only informative for stimuli in the “release” conditions, because in the “no” conditions responses were given after a fixed interval. For each participant, averages for correct Reaction Times (RTs) were calculated for each word category during LD and SD. For statistical analysis, we performed an ANOVA as well as linear mixed-effects modeling (LME) considering as within-subject factors *task* (Lexical and Semantic Decision Task), *response type* (hand motor response and foot motor response), and *word type* (arm-related and leg-related words). Compared to traditional analysis of variance, LME is a more flexible (albeit more complex) modeling approach, which allows the incorporation of subjects and items as crossed random factors (Baayen, Davidson, & Bates, 2008). In our LME model, we included intercepts as well as slopes corresponding to the fixed effects as subject-dependent random effects.^1^ LME analysis was performed in the statistical software package R (Version 3.1.3), using the functions lmer() from the package lme4 (Bates, Maechler, Bolker, & Walker, 2014), and the function summary() from package lmerTest (Kuznetsova, Brockhoff, & Christensen, 2014) to obtain beta estimates, *t*- and *p*-values.

Effect sizes were also calculated and reported as suggested by Lakens (2013).

Performance accuracy was evaluated using non-parametric tests. Differences between *tasks*, *response types* and *word types* were separately tested using Wilcoxon rank tests for related samples; while, differences within each of these conditions were assessed using Friedman's 2-way ANOVAs.

We defined as *Congruency Condition* (CC) the case of *same effector and word type* (e.g., arm-word presentation after button press by finger), and as *No Congruency Condition* (NCC) the case *of different effector and word type* (e.g., foot-word presentation after button press by finger).

### 2.7 EEG/MEG pre-processing

The temporal extension of signal-space separation (tSSS) method was applied during the pre-processing stage of analysis using Elekta Neuromag MaxFilter software, together with the detection of statically bad channels and head movement compensation (Taulu & Simola, 2006). EEG and MEG data were band-pass filtered off-line at 1–30 Hz. The continuously recorded neurophysiological data were divided into epochs of 1700 msec length, starting 700 msec before word presentation (i.e., 200 msec before the red dot presentation that indicated the beginning of a trial). Baseline correction was applied by subtracting the average response of the 100 msec prior to the red dot presentation (−700 to −600 from the onset of the word presentation) from all data points throughout the epoch. Epochs were rejected when, within −100 to 500 msec of word presentation, maximum–minimum amplitudes exceeded the following thresholds for any channel: 120 μV for EEG, 150 μV for the EOG, 3000 fT for magnetometers, 1000 fT/cm for gradiometers. Trials containing behavioral errors were also excluded from subsequent analysis.

### 2.8 Data analysis and statistics

**Fig. 2:**
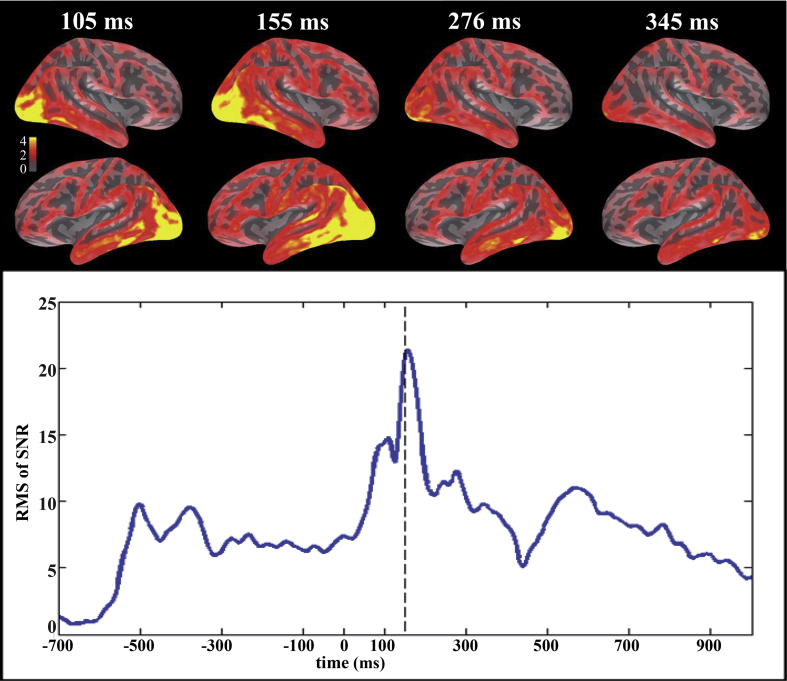
Time course of the EEG/MEG signals in sensor and source space. Source estimates for the average across all conditions at latencies derived for peaks in the time courses below. Activation is displayed on the right (top) and left (bottom) hemispheres of the inflated average cortical surface. Color scale is in nA. The time courses at the bottom represent root-mean-square (RMS) of signal-to-noise ratios (SNR) for all EEG and MEG sensors across time.

We hypothesized that pre-activation of the motor system affects activation in classical language areas and motor regions already at early stages of language processing. In order to exactly define this latency range of interest, the time course of the data was determined by means of the root-mean-square (RMS) of the signal-to-noise ratio (SNR) across all magnetometers, gradiometers and electrodes for all action-words in both tasks (see Fig. 2). The SNR was computed as the signal at a particular channel and at a particular latency divided by the standard deviation of the baseline interval of this channel (as, e.g., in Hauk et al., 2012). The computation of SNR prior to RMS makes the values for all channels unitless (original measurements are in T, T/m and V, respectively), and allows the computation of a combined measure for display.

### 2.9 Source estimation

For each participant, the brain's cortical surface was reconstructed from structural MRI images processed using automated segmentation algorithms of the FreeSurfer software (Version 4.3; http://surfer.nmr.mgh.harvard.edu/). The functional data analysis was accomplished using MNE software (Version 2.6; http://www.nmr.mgh.harvard.edu). The cortical surface was down-sampled to about 10,000 vertices, with an average distance between vertices of 5 mm. EEG and MEG sensor configurations and MRI images were coregistered based on the matching of scalp surface reconstructed from the MRI images, and about 50–100 digitized locations on the scalp surface prior to the recording session. Forward solutions for combined EEG and MEG data were computed using a boundary element model (BEM) based on a three-layer segmentation of the MRI image (inner skull, outer skull and skin surfaces, 5120 triangles), created using a watershed algorithm. The noise covariance matrices for each data set were calculated for baseline intervals of 200 msec before the beginning a trial. For regularization, the default method in MNE was used, i.e., a SNR value of 3 was specified.

Source estimates were computed for each subject and each of the conditions using unweighted minimum norm estimates (MNEs; Hamalainen and Ilmoniemi, 1994, Hauk, 2004). Sources were restricted to the cortical surface of each individual applying a loose orientation constraint (parameter value .2). This means that the source component perpendicular to the surface contributes most to the estimate, while still allowing some variation in the tangential component. The individual source estimates were morphed to the group-average brain using Free-Surfer. These grand-averages are displayed on the inflated average cortical surface of all participants/taken from the Montreal Neurological Institute (MNI) standard brain.

### 2.10 ROI analysis in source space

We performed a region-of-interest analysis for five regions, selected to cover brain areas in motor cortex (Hauk et al., 2004) as well as in areas related to semantic word processing in left perisylvian cortex (Binder et al., 2009, Bookheimer, 2002, Geschwind, 1970, Patterson et al., 2007, Vigneau et al., 2006). The spatial resolution of EEG/MEG data, in the absence of specific modeling constraints, is limited (e.g., Hauk, Wakeman, & Henson, 2011). We therefore chose regions that we considered to be separable based on our combined EEG/MEG data.

For our language ROIs, we did not have localizer recordings, and the use of fMRI coordinates from the literature is not straightforward due to the limited spatial resolution and potentially systematic mislocalization of sources in EEG/MEG. We therefore used anatomical labels provided by the FreeSurfer software, and defined ROIs around peaks of activation for the average across all stimulus conditions within those labels. The contrasts of interest used for statistical analysis are orthogonal to the average across conditions, and the numbers of trials within each condition are similar. Our selection of ROIs is therefore not biased towards a particular effect (Kriegeskorte, Simmons, Bellgowan, & Baker, 2009).

**Fig. 3:**
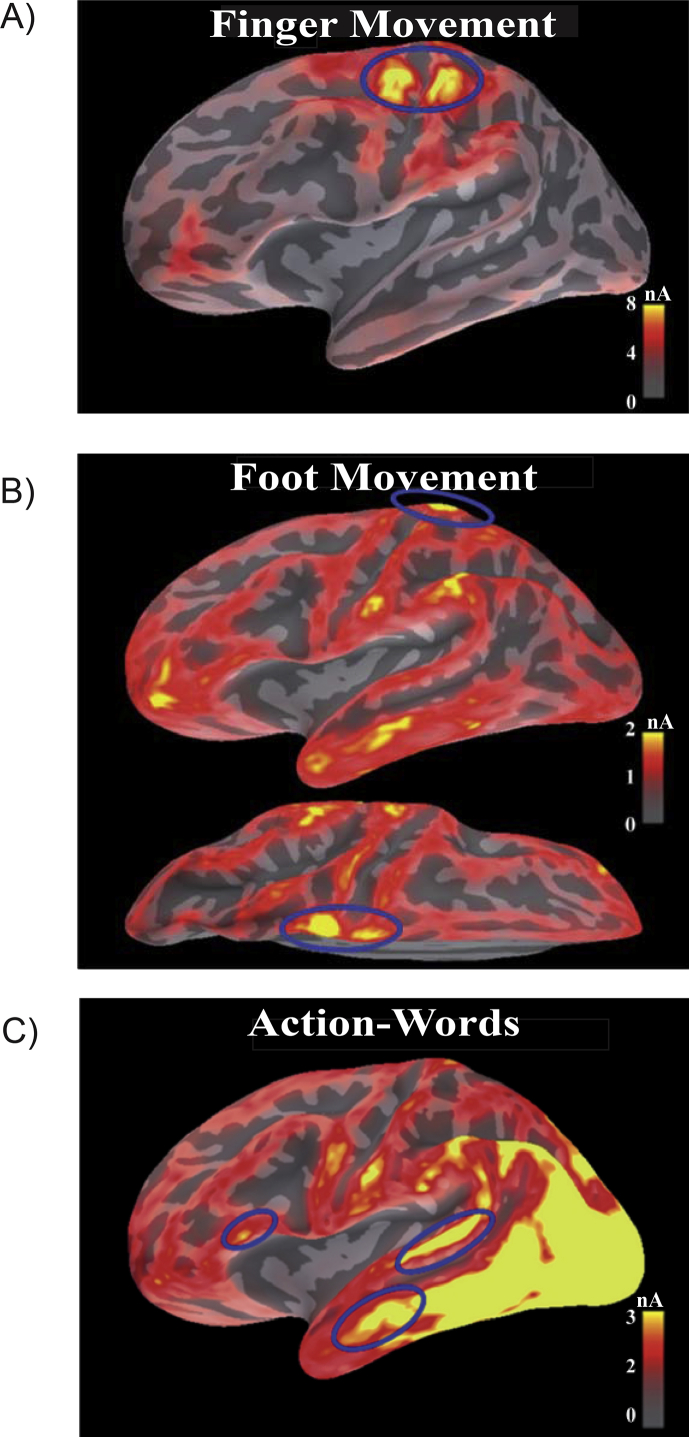
Whole-brain activation for movement types and action-words. Source estimates at peak latencies for the finger (A) and foot (B) button presses that initiated each trial, as well as for all word stimuli 150 msec after stimulus onset (C). The ellipses indicate activation peaks used to define regions-of-interest in language comprehension areas for statistical analysis.

This resulted in regions “ATL”, “pSTL” and “IFG”. We consider it likely that these regions capture activity from different parts of the brain, but it is possible that effects within each ROI also reflect “leakage” of activity from areas in their vicinity. The labeling of these ROIs should therefore not be overinterpreted. Importantly, our language ROIs are clearly separated from our motor ROIs. Our ROI selection is therefore well-suited to address our main objective, i.e., to determine to what degree pre-activation of motor areas affects language areas during word processing. The locations of our ROIs are indicated in Fig. 3.

The hand and foot *motor ROIs* were individually defined for each participant, using the source estimation results from the motor-evoked signals in the button press conditions on the group-average inflated cortex. The peak latency was determined from RMS-of-SNR curves, and ROIs were chosen around activation peaks around the vertex position (for foot) and in left central sulcus (for hand). Source estimates were visualized using the mne_analyze function in the MNE software, and the border of the ROIs were drawn approximately following the line of half peak amplitude for the corresponding activation peak. Given the sensitivity of MEG to focal, tangential and superficial source in motor cortex, we used MEG signals alone for the source estimation of the motor ROIs (Ahlfors, Han, Belliveau, & Hämäläinen, 2010). By contrast, for the more distributed activation patterns in perisylvian language ROIs, we used a combination of EEG and MEG signals for source estimation. EEG has been shown to be particularly beneficial for the detection of distributed sources (Goldenholz et al., 2009).

For the statistical analysis, mean current amplitudes were extracted for each Motor and each language ROI, respectively, within the time windows of interest identified by the sensor analysis (Fig. 3A, B and C). We tested our hypotheses of congruency motor effects separately for Motor ROIs and Language ROIs for each time interval of interest by means of 3-way repeated measures ANOVAs and LME with factors *ROI*, *response type* (hand motor response and foot motor response), and *word type* (arm-related and leg-related words). Our LME models contained by-subject random intercepts and slopes corresponding to the fixed effects. For significant interactions, we tested our directed hypothesis with respect to a congruency effects (i.e., lower amplitudes for congruent compared to incongruent response and word type pairings) using one-tailed paired *t*-tests as planned comparisons.

## 3 Results

### 3.1 Behavioral results

Response times were submitted to a repeated-measure ANOVA (“ANO”) and linear mixed-effects modelling (“LME”) with factors *task*, *response typ*e and *word type* (Table 2). Participants responded more quickly in the LD than in the SD task (LD = 673 ± 99 msec; SD = 732 ± 101 msec), as shown by a significant main effect of the factor *task* [ANO: *F*(1,16) = 19.38, *p* < .001; LME: beta = 74.9, *t* = 4.66, *p* < .001]. In addition, we found a *response type* main effect [ANO: *F*(1,16) = 8.87, *p* < .01; LME: beta = −40.7, *t* = −3.29, *p* < .01] with faster hand than foot responses (hand response: 685 msec ± 95 msec; foot response: 720 msec ± 110 msec). We did not find an interaction involving both factors *word type* and *response type*, i.e., there was no congruency effect in our behavioral data. The pattern of results did not change qualitatively when testing z-scores, median responses times, removing outliers (more than two standard deviations off the mean), or adding participants excluded from MEG analysis.^2^

Differences in performance accuracy were separately tested using Wilcoxon rank tests for related samples. Statistically significant differences were present for *task* [*W* = 118.5, *p* < .05] and *response type* [*W* = 100.5, *p* < .05]. Participants were slightly more accurate during LD (95.5% correct, SD 5.6%) than SD (92.2%, SD 4.1%). Performance was also more accurate during hand (94.5%, SD 4.3%) than foot (93.1%, SD 5.2%) responses. No difference within each task was statistically significant.

### 3.2 Functional data results

Artifact rejection led to the exclusion of 17.2% of trials on average. These exclusions can be broken down as 15.9% of trials in the LD and 18.5% in the SD task, 15.7% of the hand responses and 18.7% of the foot responses, and 16.8% of the arm-related words and 17.6% of the leg-related words. There were no statistically significant differences between the number of trials excluded for each condition (all *F* values < 1).

Fig. 2 shows the RMS of SNR curves calculated across all 102 magnetometer, 204 gradiometer and 70 EEG channels for all action-related words in all conditions, illustrating the time course of the overall signal strength of the ERP/Fs. During the first 500 msec peaks occurred at 108 msec, 156 msec, 276 msec and 342 msec after the stimulus onset. Similar to previous studies, in order to capture early short-lived effects we defined short non-overlapping time windows of 20 msec duration around those peaks (Hauk et al., 2012, Hauk and Pulvermüller, 2004, Sereno and Rayner, 2003). Although there is no consensus on the exact time course of visual word recognition, two recent studies converged on the view that semantic information retrieval can begin around 160 msec after stimulus onset (Amsel et al., 2013, Hauk et al., 2012). Differences between action-word types have been shown around 150 msec (Moseley et al., 2013) and 200 msec (Hauk and Pulvermüller, 2004, Klepp et al., 2014). Brain differences between visual object categories have been observed at 150 msec (Fabre-Thorpe, Delorme, Marlot, & Thorpe, 2001) or even earlier (Boutonnet & Lupyan, 2015). We therefore focused on our peak around 150 msec and later latencies.

We tested whether motor responses along with their associated activity in cortical motor systems affect language processing of action-related words in both motor and non-motor areas at early processing stages, and whether any such effect is specific to semantic word type. A recent study reported similar time courses for lexical and semantic decision tasks (Hauk et al., 2012), and we did not find an interaction with the factor *task* in our behavioral data. For this reason, we did not include a factor Task in the present statistical analysis of our EEG/MEG data.

In the following, we will refer to effects that involve an interaction between the factors *response type* and *word type* as “congruency effects”. Note that main effects of the factor ROI are meaningless due to differences in sensitivity, e.g., with respect to source depth, anatomy or sensor coverage. Error bars in bar graphs indicate standard errors of the mean after between-subject variability has been removed, as appropriate for repeated-measures comparisons (Loftus & Masson, 1994).

### 3.3 Perisylvian language ROIs

Non-motor perisylvian language areas were studied using an ANOVA (“ANO”) and linear mixed-effects modelling (“LME”) performed on source estimates including the factors *Language ROIs* (IFG, pSTG and ATL), *response type* (Hand and Foot) and *word type* (arm- and leg-related). The ROIs were defined based on MNE source estimates for all conditions averaged together, i.e., independently from the comparisons in the following ANOVAs (Fig. 3C).

#### 3.3.1 150 msec

**Fig. 4:**
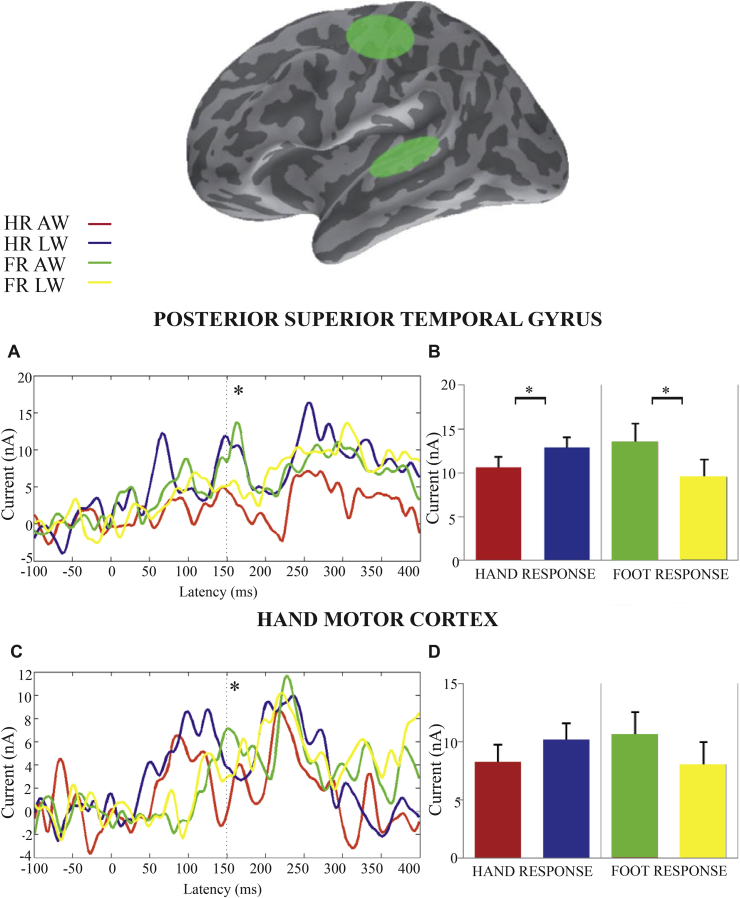
Time-course of cortical activation in the source space ROI analyses. The ellipses indicate ROIs that showed significant congruency effects for effector type (finger and foot) and word category (arm- and leg-related). A and C present the time course of activation during the four experimental conditions [hand response (HR) to arm-related words (AW), HR to leg-related words (LW), Foot Response (FR) to AW and FR to LW] in pSTG and Hand area, respectively. The zero latency marks the onset of the written word. The bar graphs B and D show the mean activation values in the corresponding ROIs in the time interval around 150 msec. Error bars indicate standard errors of the mean after individual subject variance has been removed. Asterisks indicate a significance level of *p* < .05.

There were no main effects for the factors *response type* and *word type* (*F* < 1); while there was a main effect of *Language ROIs* [ANO: *F*(2,32) = 3.77, *p* < .05, ηp² = .19, ηG² = .09; LME: beta = 7.7, *t* = 2.66, *p* < .05]. Most importantly, we found a differential congruency effect across ROIs, as shown by a statistically significant *Language ROI*(3) X *response type*(2) X *word type*(2) interaction [ANO: *F*(2,32) = 4.52, *p* < .05, ηp² = .15, ηG² = .01; LME: beta = −6.1, *t* = −1.73, *p* = .086]. More detailed analyses on each level of the Language ROIs factor revealed that only pSTG showed a significant congruency effect, i.e., an interaction of *response type*(2) X *word type*(2) [ANO: *F*(1,16) = 5.77, *p* < .05, ηp² = .36, ηG² = .02; LME: beta = −6.3, *t* = −2.87, *p* < .01]. Planned comparisons confirmed a cross-over interaction in pSTG, such that during the foot response activity was weaker for leg-related (9.5 nA) than arm-related words (13.6 nA) [*t*(16) = 2.07, *p* = .03, *d* = .46], while during the hand response we observed the reverse [*t*(16) = −2.03, *p* = .03, *d* = .45], with lower signal amplitude for arm-related words (10.7 nA) compared to leg-related words (12.9 nA) (Fig. 4B). Fig. 4A displays the time course of the cortical activation in pSTG. IFG and ATL did not show any significant main effects or interactions.

#### 3.3.2 276 msec

The repeated-measures ANOVA performed on source estimates around 276 msec showed a main effect of the *Language ROIs* factor [ANO: *F*(2,32) = 15.87, *p* < .001, ηp² = .56, ηG² = .19; LME: beta = 11.7, *t* = 4.23, *p* < .001]. No other main effects or interactions were significant (all *F* < 1). The analyses performed on each Language ROIs separately showed no further statistically significant effect.

#### 3.3.3 350 msec

Our analysis of the time window around 350 msec showed only a main effect of the factor *ROIs* [ANO: *F*(2,32) = 6.43, *p* < .01, ηp² = .33, ηG² = .13; LME: beta = 10.2, *t* = 3.5, *p* < .01], no other main effects or interactions reached statistical significance (all *F* < 1). Further analyses on each level of the Language ROIs factor failed to reveal any statistically significant effect.

### 3.4 Congruency effects in motor ROIs

Previous studies have reported differential effects of action-word processing on early brain activity in motor regions (Hauk et al., 2004, Moseley et al., 2013). We therefore analyzed our Motor ROIs for congruency effects during the same time intervals as above. However, the pre-activation of motor cortex by an actual movement is expected to induce strong sustained activation in motor cortex, which in this type of analysis may mask subtle differential modulations by action-word types.

We tested the hypothesis of an early congruency effect in motor areas by applying a repeated-measure ANOVA on source estimates in two *Motor ROIs* (hand and foot). These Motor ROIs were defined based on MNE localizations from the hand and foot button press conditions, respectively (Fig. 3A and B).

#### 3.4.1 150 msec

We obtained a significant congruency effect, reflected in a three-way interaction *Motor ROIs*(2) X *response type*(2) X *word type*(2) [ANO: *F*(1,16) = 8.57, *p* < .01, ηp² = .35, ηG² = .02; LME: beta = 6.6, *t* = 2.01, *p* < .05]. We also obtained a significant interaction Motor *ROI*(2) X *response type*(2) [ANO: *F*(1,16) = 4.23, *p* = .06, ηp² = .21, ηG² = .01; LME: beta = −6.3, *t* = −2.79, *p* < .01]. No other main effects or interactions reached significance (all *F* < 1).

In order to analyze the three-way interaction in more detail, we performed further ANOVAs on each motor ROI, including the factors *response type* and *word type*. We found a significant congruency effect in the Hand ROI, manifest as a significant interaction between *word type* and *response type* [ANO: *F*(1,16) = 5.04, *p* < .05, ηp² = .31, ηG² = .03; LME: beta = −4.5, *t* = −2.24, *p* < .05], whereby the congruent conditions produced lower motor activation than the incongruent condition. Lower signal amplitude was observed for hand responses during arm-related (8.3 nA) compared to foot related word (10.1 nA) processing as well as for foot responses during leg-related (8 nA) compare to arm-related word (10.6 nA) processing, although the planned comparisons were not significant. We found a trend that Foot Presses were followed by more activation to arm-words than to leg-words (*t* = 1.41, *p* = .09, *d* = .33). Other planned comparisons did not reach significance. Fig. 4C and D illustrates this interaction in the Hand ROI.

No significant main effects or interactions were found in the Foot ROI (*F* > 1). Note that foot motor areas are located in the medial portion of the central sulcus; therefore, signal from this region may be reduced because of the greater distance from the sensors (Hämäläinen et al., 1993, Hillebrand and Barnes, 2002). This may have led to lower statistical sensitivity in the Foot ROI. This interpretation is supported by our Fig. 3, which shows lower amplitudes in this region compared to Hand ROI.

#### 3.4.2 276 msec

We found a marginally significant interaction *response type* (2) X *word type* (2) [*F*(1,16) = 4.04, *p* = .06, ηp² = .20, ηG² = .02; LME: beta = −2.2, *t* = −1.06, *p* = .29]. We found a general higher amplitude during the hand responses to leg-related words (incongruent condition) (10 nA) compared to the other condition (hand response to arm-related words: 8 nA; foot response to leg-related words: 7.5 nA; foot response to arm-related words: 8 nA), but planned comparisons were not significant.

#### 3.4.3 350 msec

We did not find any statistically significant effects during this time interval.

## 4 Discussion

We used a novel movement priming paradigm to determine the degree to which semantic word processing is linked to sensorimotor systems. We investigated effects of pre-activation of specific movement-related brain areas on language processing, both in spatio-temporal brain dynamics and behavioral responses. We found that pre-activation of brain areas related to finger and foot movements led to decreased activation in motor cortex as well as pSTL during subsequent arm- and leg-word processing, respectively. This congruency effect between effector and word type already occurred around 150 msec, arguably the earliest stage of semantic information retrieval (Amsel et al., 2013, Hauk et al., 2012, Moseley et al., 2013, Pulvermüller et al., 2009). However, we did neither detect such a congruency effect in ATL and inferior frontal cortex, nor in our behavioral data. While our results provide support for a functional link from motor cortex to core language areas at the neuronal level, the behavioral relevance of this link is still not clear.

Some authors have suggested previously that requirements for embodiment of semantics in sensorimotor systems are that effects are elicited in an implicit task, and activate specific perceptual or motor regions at early latencies (Hauk et al., 2008, Kiefer et al., 2008). Our results fulfill these requirements for the case of action semantics, in so far as 1) participants did not have to explicitly focus their attention on action-related aspects of the stimuli; 2) effects were predicted for specific motor and non-motor brain areas; 3) effects occurred at early latencies, around 150 msec. It is still an open question whether these criteria are sufficient for embodiment, which will be discussed in more detail below.

The use of a movement priming paradigm enabled us to activate neuron populations specific to movement execution with a particular effector. This is an important advantage with respect to TMS, which may stimulate more than just the targeted area, and within this area more than just the targeted neuron population. Both methodologies are susceptible to leakage among connected brain areas. However, differential effects and double dissociations such as the congruency effect in our study provide novel evidence for effector-specific involvement of motor areas in language processing. Our results showed specific effects of motor effectors on activity in non-motor language area pSTL. When a button was being pressed using the finger prior to the presentation of an arm-word such as “stir”, brain activity in hand motor cortex and in pSTL was reduced compared to incongruent conditions (for example in response to the word “jump”). The latency of 150 msec is consistent with the earliest stages of semantic information retrieval (Amsel et al., 2013, Hauk et al., 2012, Pulvermüller et al., 2009).

We demonstrated motor-priming effects as activity reduction in motor and language areas to action words in congruent action contexts. This suggests facilitation of action-word processing in the congruent conditions. However, we were not able to find similar semantic priming effects in behavior. Most previous neuroimaging studies were not designed to reveal behavioral effects for different action-word types, which requires some sort of context or task manipulation, and their results cannot be compared to ours. Several behavioral studies have reported an interference between semantic sensorimotor features of linguistic stimuli and response execution (e.g., Boulenger et al., 2006, Glenberg and Kaschak, 2002, Liepelt et al., 2012, Meteyard et al., 2008). The strongest evidence for a causal role of sensorimotor systems in semantic processing would be a specific break-down or facilitation of semantic processing after specific impairment or stimulation of sensorimotor systems. In a single-pulse TMS study, facilitation – not impairment – of hand- and foot-word processing after hand and foot motor cortex stimulation was reported (Pulvermüller, Hauk, Nikulin, & Ilmoniemi, 2005), but Tomasino et al. (2008) did not find a comparable effect. According to Binder and Desai (2011), the evidence from lesion studies suggests that “conceptual deficits in patients with sensory-motor impairments, when present, tend to be subtle rather than catastrophic”.

Our results would undoubtedly have been stronger if a congruency effect had been obtained in behavior as well. There may be methodological reasons for the absence of behavioral effects. Our participants had to release (rather than press) a button in response to our word stimuli, after it had been pressed continuously for more than 500 msec. It is as yet unclear how small additional activation during word processing may affect this type of response. Furthermore, our button presses required very specific finger and foot movements, which may only partly have overlapped with the actions denoted by our word stimuli (see Appendix). The corresponding priming effects may therefore have been too weak to be detected. While the neuronal specificity of the pre-activation is one of the advantages of our movement priming paradigm, it also poses novel challenges on stimulus selection and experimental design, which should be addressed in future studies.

We would like to note that we did not find reliable congruency effects after 150 msec either. It may be the case that our subtle effects are indeed specific to early latency ranges, but word recognition “catches up” at later latencies. This may be related to the absence of behavioral priming effects in our study. A similar point has recently been made with respect to task effects in visual word recognition, which appeared at early but not later latencies (Chen, Davis, Pulvermüller, & Hauk, 2015). A more detailed analysis of the time course of movement priming effects and its relevance for behavior must be left for future studies. In the present study, we made the first attempt to link early brain responses with behavior in a movement priming paradigm. We could confirm and complement previous findings on the time course of semantic processing, but failed to find a clear link between brain responses and behavior. We consider it as essential that future research investigates the link between behavioral and brain responses in more detail. Our movement priming paradigm will be a valuable tool in this endeavor.

The core finding of the present study is the specific activity reduction for the congruent conditions in pSTL. The spatial resolution even of combined EEG and MEG source estimates is inherently limited, which restricts the interpretation of our results with respect to precise anatomical areas comparable to fMRI analysis (Goldenholz et al., 2009, Hauk et al., 2011, Molins et al., 2008). Brain areas around pSTL have consistently been linked to language comprehension, in neuropsychological as well as neuroimaging studies (“Wernicke's area”, e.g., Binder et al., 2009, Geschwind, 1970, Price, 2000). An involvement of this region in language processing has also been demonstrated using priming paradigms (Helenius et al., 1998, Nobre et al., 1994, Simos et al., 1997). The typical brain activity reduction following the presentation of a word primed by a semantically related word has been reported in superior temporal lobe both during semantic and phonological priming tasks (Kujala et al., 2012, Vartiainen et al., 2009). This ROI was therefore likely to show a semantic congruency effect between effector type and word category in language comprehension, as confirmed by our results.

We did not find effects in ATL and IFG. Note that we used combined EEG and MEG measurements, which maximized the sensitivity of our measurements (Goldenholz et al., 2009, Molins et al., 2008). Inferior frontal areas have been implicated in semantic processing by some authors (Bookheimer, 2002, Poldrack et al., 1999). Nonetheless, these areas are more frequently associated with phonological and syntactic processing as well as response selection (Binder and Desai, 2011, Grodzinsky and Friederici, 2006, Jobard et al., 2003, Marslen-Wilson and Tyler, 2007, Thompson-Schill, 2003). Furthermore, our Fig. 3 shows that IFG produced the least amount of overall activation among the perisylvian ROIs in the present experiment. The null effect in this ROI is therefore not surprising.

We would still have expected a congruency effect in ATL, as this area has been labeled as a “semantic hub” in the hub-and-spoke model of Patterson and Rogers based on neuropsychological and neuroimaging findings (Patterson et al., 2007, Pulvermüller et al., 2010, Rogers et al., 2006). fMRI studies have reported semantic priming in ATL (Gold et al., 2006), and masked semantic priming has been reported in a recent MEG study, although in the N400 latency range (Lau, Gramfort, Hämäläinen, & Kuperberg, 2013). Our null result cannot be taken as evidence against ATL's possible role as a semantic hub, as it may reflect a lack of statistical sensitivity, e.g., due to incomplete sensor coverage.

In conclusion, this experiment added new evidence to the growing literature that the motor system contributes to semantic processes in the human brain. We confirmed that action-related stimuli activate motor cortex early on in processing. Importantly, this early effect depended on the congruency between the effector used for pre-activation of motor areas and the meaning of subsequently presented action-words. In contrast to many previous neuroimaging studies, our movement priming paradigm allowed testing for a directional link from motor to language systems. However, we did not find a congruency effect on behavioral responses, raising questions about the behavioral relevance of our and previous neuroimaging results. We hope that our movement priming paradigm, in combination with the high temporal resolution and optimized spatial resolution of combined EEG and MEG measurements, will prove useful to further clarify the link between brain and behavior for semantics as well as in other cognitive domains.

## Figures and Tables

**Table:** 

Arm-words	Leg-words	Pseudowords	Abstract words
braid	crawl	benk	abate
carve	creep	berm	admire
chop	cycle	blant	adore
clap	flee	blash	amaze
clean	Flit	blean	annoy
click	glide	bline	assess
cut	hobble	boit	betray
darn	hop	breal	bid
delve	jive	cade	bide
draw	Jog	cauth	bode
dunk	kick	cheel	bore
file	kneel	chemp	botch
fold	lame	chib	calm
frisk	leap	chont	cease
grasp	limp	churm	cheat
grate	march	cloap	cloy
grip	mount	clode	coax
grope	pace	craff	cope
hack	plod	crish	crave
knead	race	crite	dare
knit	roam	crusk	desire
peel	run	dage	devise
pinch	scuff	dince	dream
poke	skate	doint	dupe
scoop	skip	dreen	dwell
scrape	skulk	drell	earn
scratch	slide	drosh	envy
scrub	slink	dulge	exalt
seize	sneak	durve	excite
shape	step	filk	fancy
skim	stomp	flike	faze
slash	stray	flosh	feign
stab	stroll	fush	fend
stir	strut	glist	flirt
stroke	stumble	goll	focus
tap	tread	hust	glean
touch	trip	jang	glut
whip	trot	jant	goof
wipe	trudge	jate	grieve
write	walk	jick	hate
		larp	haunt
		lunk	heal
		mib	hope
		mish	invent
		mive	irk
		murse	jib
		narty	lapse
		nask	laze
		nate	learn
		nipe	love
		pank	lure
		pesk	marvel
		plounce	mope
		pluff	mull
		ponk	muse
		posk	opine
		prolt	owe
		proost	pall
		prute	ponder
		quish	quell
		ralve	recall
		rast	reckon
		rosk	relax
		shonk	sadden
		slinch	sate
		smirch	scorn
		snate	skimp
		sprode	slake
		stent	solve
		sweel	spare
		tect	spurn
		traft	stash
		traut	stun
		trenk	tempt
		tunk	think
		verm	trust
		vink	waive
		wump	wish
		yoss	worry
		ziss	yearn

**Table 1:** Stimulus properties.

		Word frequency	Orthographic neighborhood size	Bi-gram frequency	Tri-gram frequency
Arm-related words	4.6	21.4	6.5	1152.4	135.1
Leg-related words	4.6	21.8	6.5	1039.2	163.3
Pseudowords	4.7	/	6.3	1145.9	149.302
Abstract words	4.7	30.3	5.8	1265.1	191.8

Psycholinguistic parameters for all stimulus categories. Frequencies are reported as per million.

**Table 2:** Mean Reaction Times in LD and SD (in msec).

Task	Lexical decision task		Semantic decision task	
Response type	Hand	Foot	Hand	Foot
Arm-related words	655	694	713	757
Leg-related words	656	685	708	744

## Footnotes

1 Using R notation: data ∼ V1*V2 + (1 + V1 + V2|subjects) + (1|items).

2 These analyses were suggested by an anonymous reviewer.
